# The effect of a controlled manipulation of maternal dietary fat intake on medium and long chain fatty acids in human breast milk in Saskatoon, Canada

**DOI:** 10.1186/1746-4358-5-3

**Published:** 2010-02-19

**Authors:** Roseann Nasser, Alison M Stephen, Yeow K Goh, M Thomas Clandinin

**Affiliations:** 1 University of Saskatchewan, College of Pharmacy and Nutrition, Saskatoon, Saskatchewan, Canada; 2 Alberta Institute for Human Nutrition, Edmonton, Alberta, Canada; 3 MRC Human Nutrition Research, Elsie Widdowson Laboratory, Cambridge, UK; 4 Regina Qu'Appelle Health Region, Saskatchewan, Canada

## Abstract

**Background:**

Few studies in recent years have demonstrated the effect of maternal diet on fatty acid composition of human milk.

**Methods:**

Fourteen free-living lactating women participated in a cross-over dietary intervention study, consuming a low fat diet (17.6% of energy as fat, 14.4% of energy as protein, 68.0% of energy as carbohydrate) and a high fat diet (40.3% of energy as fat, 14.4% of energy as protein, 45.3% of energy as carbohydrate) each for periods of 4 days, in randomised order. Each mother was her own control. Mature milk samples were collected during each period and analysed for medium and long chain fatty acids.

**Results:**

The concentration of medium chain fatty acids (MCFA), was 13.6% in breast milk for the low fat diet compared to 11.4% for the high fat (p < 0.05). Arachidonic acid (C20:4n-6) levels were significantly higher in breast milk when women consumed the low fat diet. Increased dietary intake of stearic acid (C18:0) and alpha-linolenic acid (C18:3n-3) on the high fat diet significantly increased proportions of these fatty acids in breast milk (p < 0.05) in 4 days.

**Conclusions:**

Changing maternal dietary fat intake has a rapid response in terms of changes to fatty acids in breast milk.

## Background

Maternal diet has been shown to influence fatty acid composition of breast milk, with changes appearing within 8-10 hours after a meal is consumed [1-3]. Fatty acids with a chain length greater than C14:0 originate from the maternal diet or body stores, while fatty acids up to C14:0 originate from *de novo *synthesis in the breast [1,4].

There is a paucity of randomised controlled dietary intervention studies and cross-over designs where all meals have been provided to lactating women for each time period using precise food consumption measures, a combination of dietary fats, and proportions of dietary fat that could be achievable in normal living over short time periods. Harzer *et al*. investigated the effect of diet on three women who had been breast feeding for more than six months. These women were asked to prepare their own meals and consumed the diets for two weeks [5]. While Insull *et al.'s *influential study was conducted with one woman who was nine days postpartum where she was provided with seven consecutive feeding periods of at least four days duration over several weeks and no wash-out period [6]. Other investigators have demonstrated the effect of controlled diets on small samples of lactating women [5-7] using controlled diets with 5% dietary fat [7,8], testing individual fats or groupings of fat [9-11], dietary fat in vegetarian lactating women [12], and in observational studies [1,13-15].

The purpose of this study was to determine the effect of short term dietary manipulations of low and high fat diets on the milk fat content and fatty acid content of mature human breast milk in a sample of free-living Canadian women, and specifically, to determine the effect of dietary fat content on medium chain fatty acids (MCFA), and long chain fatty acids (LCFA) linoleic, α-linolenic, docosahexaenoic and arachidonic acids in mature breast milk.

## Methods

### Inclusion criteria

To be eligible to participate in this study, healthy women, not taking any prescribed medications, between the ages of 18-40 years of age, who were exclusively breastfeeding infants between 2-6 months of age were invited to participate. Vitamin and mineral supplements were allowed. Women could not be vegetarian and had to eat all the items on the menu provided to them. Invitations to take part in the study were posted in offices of general practitioners and in public health satellite units.

Fourteen healthy women living in Saskatoon, Canada met the inclusion criteria and volunteered to participate in the study. This study was conducted in 1995-1996. All participants provided written informed consent. The study procedure was approved by the University of Saskatchewan Advisory Committee for Ethics in Human Experimentation.

### Usual diet

Prior to taking part in the period of diet control, the women recorded everything they ate and drank for a period of three days, of which two days were weekdays and one a weekend day, to take account of different eating patterns on the weekend versus during the week [16]. To ensure completeness and accuracy of dietary records, the research team reviewed the dietary records with the participants. The three-day diaries were coded and analyzed for nutrients using the Nutritional Assessment System (NUTS) program [17].

### Dietary intervention

The study was a cross-over design with randomised order of the participants. The women were randomly assigned to receive each dietary intervention (low fat and high fat) for four days each with three day wash-out period, with equal numbers of participants for each order of treatment. The women were unaware of the dietary assignment. Each woman served as her own control. The women consumed their own diet prior to and during the three-day wash-out period between the two controlled dietary test periods. For the two test periods, women were provided with prepared meals and snacks meeting their daily energy requirement, as determined by the dietary assessment prior to the period of diet control. Meals were prepared in the Division of Nutrition and Dietetics Metabolic Unit and delivered daily to the women's homes. The diets were designed in a two-day rotating menu and were made up of typical North American foods. A number of foods were the same on each of the two days, with lunch and dinner having different food items. The low and high fat diets differed only in specific foods, such as milk, margarine, salad dressing, cheese, fish, ice-cream, soft drinks, cookies and cake, where high fat versions were used for the high fat diet, and low fat versions for the low fat diet. To ensure equivalent energy intake, the low fat diet had a higher carbohydrate level, achieved by altering cake and cookie recipes, using a carbohydrate source, Accugel (Woodstone foods, Winnipeg), derived from pea starch, and by adding specific high carbohydrate items, like jelly beans, and regular soft drinks. The low fat diet had a macronutrient composition of 17.6% energy from fat, 14.4% energy from protein and 68.0% energy from carbohydrate, the high fat diet 40.3% energy from fat, 14.4% energy from protein and 45.3% from carbohydrate.

Fatty acid composition of test diets was calculated using USDA Handbook No.8 and the Canadian Nutrient File (Table 1). For both diets, saturated fatty acids were the main contributor, with lower, but similar proportions of monounsaturated fatty acids (MUFA) and polyunsaturated fatty acids (PUFA). Since changes in fat content are most easily achieved by providing different fat levels of dairy products, like milk, cheese, and ice-cream, total saturated fatty acids represented 41% of total fatty acids for the high fat diet and 34% of total fatty acids for the low fat diet. As a result, dietary PUFA were present in higher proportions for the low fat diet compared to the high fat. The proportion of α-linolenic acid was approximately the same on the low and high fat diets (2.99 and 3.15%, respectively), while the proportion of docosahexaenoic acid was about 2.5 times higher on the low fat diet than the high fat diet (0.54 and 0.22%, respectively). Linoleic acid consumption on the high fat diet was more than 70% as much on the low fat diet (19.78 and 11.57 g/d, respectively), but in proportional figures it was considerably lower: 21.92 versus 28.99% of total fatty acids.

**Table 1:** Calculated mean dietary intake (two-day) of fatty acids in the test diets (g/day)

		Fatty acid intake (g/d)	
	
	Low fat		High fat
**Total fat**	46.30		104.60
**Total fatty acids**	39.90		90.21
**Saturated fatty acids**	15.76		42.84
C4:0	0.60		1.75
C6:0	0.26		1.75
C8:0	0.18		0.58
C10:0	0.42		1.30
C12:0	0.56		1.66
C14:0	1.87		5.75
**Total MCFA (C10:0-C14:0)**	2.85		8.71
C16:0	8.67		21.74
C18:0	3.20		8.31
**Total MUFA**	10.90		24.38
C16:1	1.00		2.04
C18:1	9.84		22.18
C20:1	0.04		0.11
C22:1	0.02		0.05
**Total PUFA**	13.24		22.99
C18:2	11.57		19.78
C18:3	1.29		2.72
C20:4	0.06		0.08
C20:5	0.06		0.08
C22:5	0.04		0.13
C22:6	0.22		0.20

The diet was designed with a basal energy of 2155 kcal for the low fat diet and 2192 kcal for the high fat diet. If a woman had higher or lower energy requirements, increments were added or taken away. Increments were made up of particular food items such that fat, protein and carbohydrate components were in the same proportions as in the basal diet. The energy given to each woman during the period of diet control was based on average energy intakes assessed from three-day food diaries. Past experience of controlled energy intakes in healthy individuals in our laboratory suggests that three-day records along with questioning about usual diet, provides a good assessment of intake to maintain body weight. It was important for the women to not change their energy balance from one four-day period to the next. Women who breastfeed have more variable demands than other subjects, and some variation was permitted. Slight energy adjustments were made, if necessary, between the two periods. It was expected that the women would eat all the food supplied to them. Participants of this study were counselled to follow the diet to ensure adherence and the importance of consuming only what was provided. Compliance was measured each day at the time of the home visit. Each woman was provided with a food item checklist that was reviewed daily by the researchers. Each woman was also interviewed daily and at the end of each four-day period regarding adherence to each diet.

### Milk collection

The first two days of each four-day period were considered an equilibration period and milk samples were provided on the second two days. Each mother expressed 20-50 mL of breast milk on the last two days of each four-day period between 1-2 p.m., using a manual Ameda Egnell breast pump [18-21]. Women were instructed on how to use the pump and when to collect the milk. They were instructed to have their baby latch to the breast and after 2-5 minutes of feeding, they were to latch the child onto the other breast while pumping milk from the first breast. Unlike the total fat content of milk, milk fatty acid composition does not change during a feed or from breast to breast [21,22]. The pumped milk samples were immediately transferred into sterilized containers with caps and placed into the home freezer (-10 to -20°C) until the research team collected them later that day, half an hour to two hours following collection. Milk samples were kept cold during transport and stored in a -70°C freezer until analysis [23].

### Medium and long chain fatty acid analysis

Breast milk samples were extracted for medium and long chain fatty acids using a modified Folch procedure [22,24]. One mL of breast milk was extracted with 20 mL chloroform and methanol (chloroform:methanol 2:1) and 4 mL 0.05% CaCl_2_. Santoquin, an anti-oxidant was added. After separation, 1 mL of the lower chloroform phase was removed and dried under nitrogen. One mL 0.5 M KOH was added and the mixture heated for 1 hour at 110°C. On cooling, 1 mL boron trifluoride (BF_3_) in methanol (14% w/w) and 2 mL hexane was added and heated off to 110°C for 1 hour. On cooling, 2 mL water was added and samples stored cool over night. The top layer was removed, dried, made up with 100 μL hexane and stored at -70°C until analysis. The average of the two days was reported for fatty acid concentrations.

### Gas chromatography parameters

A Varian Vista 6000 GC with a Varian Autosampler 8000 and Star Chromatography Workstation Version 4.0 program was used to analyse these samples. The column was an SGE BP-20 capillary column (25 m × 0.22 mm i.d., 0.25 μm film). Temperature was programmed at 75° to 165° at 20° per minute, then 165° to 220° for 13 minutes and at 220° for 6 minutes. Injector and detector ports were set at 250° and 270° respectively. The split ratio was 100:1. Helium was the carrier gas with a linear velocity of 30 cm/sec. The results were expressed as g fatty acid/100 g of fat. The following fatty acids were quantified: C10:0, C12:0, C14:0, C16:0, C18:0, C20:0, C14:1n-9, C16:1n-7, C18:1n-9, C18:1n-9, C18:1n-9, C18:2n-6, C18:3n-3, C18:3n-6, C20:3n-6, C20:4n-6, C22:5n-3, C22:6n-3.

### Total fat content of milk

The remainder of the lower chloroform phase from the Folch procedure was dried under nitrogen and weighed, allowing calculation of percent fat from the 1 mL sample originally taken [25].

### Statistics

All samples were run in duplicate. The results from the two days of sampling in each period were averaged for the concentrations of medium chain fatty acids (MCFA), and long chain fatty acids (LCFA) in the breast milk, total fat content and the composition of fatty acids in the diet. Since each woman was her own control and the order of treatments was randomised, with equal numbers in each group, paired t-tests were used to compare the composition of breast milk between the two diet treatments, using SPSS Version 16.0 For Windows (SPSS Inc, Chicago, IL). Fatty acids results are expressed as mean ± SEM.

## Results

Fourteen women aged 24-37 years (mean 31.6 y) with a mean BMI of 26 kg/m^2 ^participated in the study. All were healthy and non-smokers; six were primiparae and eight were multiparae. All babies were born at term with average age at the time of the study being 2.8 ± 0.9 (SD) months. Usual energy intake, as assessed using the 3-day diaries ranged from 6.4-13.6 MJ/d (1542-3265 kcal/d) with an average of 9.9 ± 1.9 MJ/d (2364 ± 472 kcal/d) (mean ± SD). The average macronutrient intakes as % energy were: fat 34%, protein 15% and carbohydrate 51%, with ranges: fat (20.6-46.0%), protein (11.3-19.1%) and carbohydrate (44.0-64.5%).

During the test periods, average energy intake for the low fat period was 9.9 ± 0.9 MJ/d (2369 ± 228 kcal/d) and 10.1 ± 1.1 MJ/d (2422 ± 261 kcal/d) for the high fat period. The average energy intake for both periods was not significantly different from the assessed habitual energy intake.

### Fatty acid composition of breast milk

Mean concentrations of breast milk fatty acids for the two test periods are shown (Table 2). There were no significant changes in the mean total concentrations of total saturated fats, monounsaturated fats and polyunsaturated fats, however, the concentration of total MCFA (C8:0-C14:0) was significantly higher when consuming the low fat diet at 13.6 ± 0.7% versus 11.4 ± 0.9% on the high fat diet (p = 0.01). Of the MCFA, the concentration of capric acid (C10:0) was significantly higher when consuming the low fat diet at 0.9 ± 0.04% than 0.7 ± 0.03% on the high fat diet (p = 0.01); and lauric acid (C12:0) was significantly higher when consuming the low fat diet at 5.4 ± 1.2% versus 4.0 ± 0.4% (p = 0.01). In addition, of the LCFA, palmitoleic (C16:1n-7) and arachidonic acids (C20:4n-6) were significantly higher on the low fat diet (p = 0.046 and p = 0.02 respectively). Stearic acid (C18:0) and α-linolenic acid (C18:3n-3) were significantly higher when women consumed the high fat diet (p = 0.01 for both). Of note, other fatty acids such as γ-linolenic acid (C18:3n-6) and dihomo-γ-linolenic acid (C20:3n-6) were significantly higher on the low fat diet (p = 0.02 and p = 0.03 respectively).

**Table 2:** Effect of changing fat content of maternal diet on MCFA and LCFA in breast milk (mean ± SEM) (n = 14)^a^

Fatty acid (g/100 g total fatty acids)	Low Fat	SEM	High Fat	SEM	p value*
**Total saturated fatty acids**	41.1	0.82	40.40	0.85	0.46
Caprylic acid (C8:0)	Trace		Trace		
Capric acid (C10:0)	0.87	0.04	0.68	0.03	0.01*
Lauric acid (C12:0)	5.38	1.16	3.98	0.37	0.01*
Myristic acid (C14:0)	7.31	0.35	6.76	0.48	0.07
**Total medium chain fatty acids**	13.56	0.66	11.42	0.86	0.01*
Palmitic acid (C16:0)	22.70	0.45	23.43	0.24	0.43
Stearic acid (C18:0)	5.00	0.11	6.08	0.14	0.01*
Arichidic acid (C20:0)	0.08	0.07	0.12	0.01	0.02*
**Total monounsaturated fatty acids**	38.70	0.80	39.90	0.65	0.10
Myristoleic acid (C14:1n-5)	0.35	0.01	0.41	0.05	0.27
Palmitoleic acid (C16:1n-7)	1.95	0.29	1.31	0.23	0.05 (0.046)*
Oleic acid (C18:1n-9)	36.16	0.86	37.81	0.67	0.08
Eicosenoic acid (C20:1n-9)	0.27	0.02	0.38	0.02	0.01*
**Total polyunsaturated fatty acids**	16.90	0.66	16.40	0.48	0.54
Linoleic acid (C18:2n-6)	14.65	0.62	13.82	0.45	0.27
α-linolenic acid (C18:3n-3)	1.22	0.04	1.69	0.06	0.01*
γ-linolenic acid (C18:3n-6)	0.12	0.01	0.09	0.01	0.02*
Eicosadienoic acid (C20:2n-6)	0.19	0.02	0.18	0.01	0.51
Dihomo-γ-linolenic acid (C20:3n-6)	0.27	0.02	0.24	0.02	0.03*
Arachidonic acid (C20:4n-6)	0.34	0.01	0.30	0.02	0.02*
Eicosapentaenoic acid (20:5n3)	Trace		Trace		
Docasapentaenoic acid (C22:5n-3)	Trace		Trace		
Docosahexaenoic acid (C22:6n-3)	0.12	0.02	0.14	0.04	0.77

^a^2-day mean of fatty acid content; *p < 0.05

### Fat content of the milk

The average fat content of hind milk for the low fat period was 5.4 ± 0.4% (SEM) and for the high fat 5.7 ± 0.4% (p = 0.54). Since this analysis was conducted on one sample during a feed, the values cannot be considered to be representative of the fat content of the entire feed or of the milk production for the day.

## Discussion

This study investigated the effect of physiologic dietary fat intake of free-living Canadian lactating women on the fatty acid composition of medium and long chain fatty acids in mature breast milk. Using a combination of dietary items reflective of the Canadian diet, women were provided with all their food and energy requirements for two four-day periods. The investigators were confident that the women complied well with the diets as there was continuous interchange and interviews, with an understanding of the importance of consuming only what was provided. In addition, others have demonstrated that participant compliance in controlled dietary intervention studies is typically high [26].

The fat content of the hind milk samples was constant during both dietary periods which is consistent with Jensen [20]. In the present study, breast milk MCFA concentrations were significantly higher when the low fat, high carbohydrate diet was consumed. Since MCFA intake in g/day was higher in the high fat than in the low fat period, these significant changes in MCFA concentrations suggest that *de novo *fatty acid synthesis may be altered rapidly when the fat content of the maternal diet is changed and that four days is indeed a long enough period to assess changes in breast milk fatty acid composition [2,3]. The magnitude of the change of MCFA from the present study is small compared to the higher MCFA content in breast milk of women in Africa, whose diets are low in fat and rich in carbohydrates [13]. It can be expected that during fatty acid analysis there is a higher loss of the shorter-chain MCFA (C8-10) as compared to the longer-chain ones (C12-14) [22]. Medium chain fatty acids are important in breast milk as they are more easily absorbed than long chain saturated fats as MCFA do not require the carnitine transport process [27].

Like Francois *et al*. and Mellies *et al*. we found no change in the fatty acid of palmitic acid in breast milk, even though the amount ingested on the high fat diet was 2.5 times higher than on the low fat diet [9,28]. The significant increase of arachidonic acid (C20:4n6 or AA) in breast milk when women consumed the low fat diet is unexpected and difficult to explain. There appeared to be no change in linoleic acid (C18:2n-6) in breast milk, albeit the increased consumption of dietary linoleate from the low fat period at 11.6 g/d to 19.8 g/d on the high fat period (respectively 4% and 7% of total dietary energy). This is unexpected, as the consumption of this fatty acid has been shown to raise the milk content of linoleic acid [1,6,9]. In these earlier intervention studies, however, the higher absolute linoleate was associated with higher relative intake as well, whereas in the present study - due to the type of fat modification - the proportional linoleic acid intake was lower on the high fat diet compared to the low fat diet (21.92% versus 28.99% of total fatty acids). Of note in the present study, both γ-linolenic acid (C18:3n-6) and dihomo-γ-linolenic acid (C20:3n-6) were significantly higher on the low fat diet, which is interesting as the former is the desaturation product of linoleic acid and the latter is the elongation product of γ-linolenic acid (C18:3n-6).

In contrast, both stearic (C18:0) and α-linolenic acid (C18:3n3 or ALA) were significantly higher in the breast milk when the high fat, low carbohydrate diet was consumed (Table 2). Francois *et al*. observed that milk content of stearic acid increased 14 hours after ingestion of a single dose of cocoa butter [9], while others noted no change in this fatty acid after the ingestion of a diet high in saturated fats over a four week period [28]. The increase in stearic acid may be a reflection of the higher proportion of dietary stearic acid when consuming the high fat diet which was two times higher than in the low fat diet.

There is much interest in whether α-linolenic acid (C18:3n3 or ALA) dietary intake alters docosahexaenoic acid (C22:6 or DHA) levels [1,29]. A high correlation has been shown between α-linolenic acid in breast milk and maternal diet [9,10]. In the present study, dietary α-linolenic acid was 1.29 g/d and 2.72 g/d (0.5% and 1.1% of total energy) when consuming the low and high fat diet respectively. The higher α-linolenic intake during the high fat period significantly increased the proportion of α-linolenic acid in the milk, yet there was no significant change observed in DHA levels. These findings were expected given the short duration of the high fat diet intake [9,10]. The regular consumption of foods high in DHA is more likely to increase DHA levels in human milk [1,30,31].

## Conclusions

In conclusion, the present study was a prospective cross-over dietary intervention where each woman was randomised to receive both diets and served as her own control. This study demonstrates the acute effects of a physiologic low fat and high fat diet on the fatty acid composition of mature human breast milk (≥ 2 months) of a group of free-living Canadian women. Although the absolute changes in the MCFA were small, the changes do suggest that *de novo *synthesis may be increased in four days when lactating women consume a low fat, high carbohydrate diet. In addition, stearic acid and α-linolenic acid may also be altered in four days in breast milk and may be reflective of maternal high fat diet. The amount of dietary α-linolenic acid was not enough to impact docosahexaenoic acid level in breast milk in a short time frame, while arachidonic acid increased in the breast milk when a low fat diet was consumed. Changes in breast milk fatty acid composition can be achieved within a short period in time by changing dietary fat content. In circumstances where high medium chain fatty acids would be desirable for infants experiencing malabsorption or feeding intolerances, changing the maternal diet to one low in fat can have immediate beneficial consequences.

## Competing interests

The authors declare that they have no competing interests.

## Authors' contributions

AMS and RN conceived, developed and conducted the project. RN and YKG conducted the fatty acid and statistical analyses and MTC interpreted the data. RN, AMS and MTC prepared and revised the manuscript and all authors read and approved it.
